# Digital soil mapping in support of voluntary carbon market programs in agricultural land

**DOI:** 10.1371/journal.pone.0327895

**Published:** 2025-09-02

**Authors:** James R. Kellner, Christian Clanton, Kirk M. Demuth, Mitchell Donovan, Y. Katherina Feng, Mage Khim-Young, Julia Maddalena, Rose Rustowicz, David Schurman

**Affiliations:** 1 Perennial Climate Inc., Boulder, Colorado, United States of America; 2 Institute at Brown for Environment and Society, Brown University, Providence, Rhode Island, United States of America; 3 Department of Ecology, Evolution and Organismal Biology, Brown University, Providence, Rhode Island, United States of America; University of Ferrara, ITALY

## Abstract

Voluntary carbon market (VCM) programs in agriculture depend on accurate measurements of soil organic carbon (SOC) that can be deployed at scale efficiently, but barriers are preventing widespread adoption. To overcome these challenges, we developed a digital soil mapping (DSM) framework driven by machine-learning and numerous spatial covariates, including long-term climate proxies, short-term climate and weather-related variables, topographic and edaphic measurements, and remote sensing time-series summaries. We show that the model can predict SOC content in the top 30 cm of soil using 5,230 measurements of SOC in agricultural land within 47 states in the contiguous United States (CONUS). Model predictions closely matched independent measured values. The intercept and slope of the cross-validated relationship at the agricultural field level were −0.179 and 1.095. The coefficient of determination was *R*^2^ = 0.811, and the RMSE was 0.041. In contrast, comparison of independent field measurements to four publicly available SOC data products using 165 fields that contained 3,285 in-situ soil samples showed poor ability of existing public SOC maps to reproduce measured values, underscoring the importance of quantification technologies developed specifically for agricultural land and with recent soil measurements. Three prior SOC data products underestimated SOC content at small values and overestimated it at large ones, while one underestimated SOC content at all values examined. Analysis of feature importance showed that time series summaries from Sentinel-2 are the strongest predictors, followed by temperature variables and features related to surface hydrology. These findings underscore the value of geographically representative training and validation data for quantifying SOC content in agricultural land and demonstrate that feature engineering can increase the sensitivity of SOC quantification to optical remote sensing summaries. Data-driven algorithms can generate accurate estimates of field-level SOC content in agricultural land in CONUS that overcome barriers to scale in the VCM.

## 1. Introduction

Soil organic carbon (SOC) plays an important role in the voluntary carbon market (VCM) due to its ability to mitigate and offset greenhouse gas (GHG) emissions through implementation of regenerative agricultural practices [1]. Activities such as cover cropping, reduced tillage and changes to crop rotation increase SOC storage and remove GHG from the atmosphere [2]. The VCM incentivizes these practices by paying for GHG reductions and removals, which can be used to offset emissions elsewhere or sold on a secondary market.

A key challenge to the development of a robust VCM in agricultural soils is quantification. Current methods are uncertain and expensive to scale. Direct measurement, such as in-situ soil sampling, is locally accurate and can be extrapolated to larger regions [e.g., 3], but may require large sample sizes to overcome spatial variation [4–6]. An alternative to direct measurement is biogeochemical simulation. Simulation models forecast SOC sequestration using physical, chemical, and biological principles, but they are difficult to deploy at scale and in regions without a long history of academic research, including small-holder farms and crop types other than globally significant row crops [7–9].

Digital soil mapping (DSM) is able to overcome these measurement challenges. DSM refers to the practice of using empirical statistical and machine learning models to predict SOC content by associating in-situ soil samples with spatial covariates, including climate proxies, weather, soils, topography, and remote sensing data [10–14]. This approach is similar to methods used to estimate aboveground forest carbon in VCM programs [e.g., 15–18]. Numerous studies have shown that environmental and remote sensing variables can map SOC content in soils [e.g., 11,14].

Two criteria necessary for the success of the VCM in agricultural soils are the accuracy of area-based summaries and sensitivity of predictions to variables related to on-the-ground farm practice. VCM programs credit SOC sequestration within areas that can be hundreds of hectares or larger, requiring measurement techniques to be validated at the unit of agricultural land management, which may be the individual field, ranch, or collections of management units that include hundreds to thousands of individual parcels. Sensitivity to management practices is necessary to ensure that changes in SOC are attributable to changes in land management – a requirement of VCS carbon programs [19–21].

Here we develop the Advanced Terrestrial machine-Learning Analysis System for Soil Organic Carbon (ATLAS-SOC). ATLAS-SOC is a data-driven DSM framework to quantify SOC content in the top 30 cm of soil in non-tree row crops in the contiguous United States (CONUS) for use in VCM programs. We design time-series features that increase the sensitivity of remote sensing variables to SOC prediction using high-resolution satellite data from Sentinel-2 and other environmental covariates [12]. Model performance is evaluated with a geographically dependent cross-validation at both sample and field levels and benchmarked against existing publicly available SOC data products. Our analysis shows that publicly available SOC data products perform poorly when validated against recent in-situ soil samples, whereas ATLAS-SOC produces accurate estimates of field-level SOC content, underscoring the potential of data-driven SOC quantification in support of robust VCM programs in agricultural soils.

## 2. Materials and methods

### 2.1. In-situ soil samples

Data-driven calculation of SOC depends on the relationship between independently measured variables and SOC content from in-situ soil samples. SOC content is the amount of SOC in a given soil sample expressed as a percentage of oven-dry mass. The approach developed here allows in-situ soil samples collected at different locations, depths below the surface, and times to be used for model calibration and validation. It does this by using locally measured covariates generated at times close to sample collection, and by treating depth as a continuous covariate feature [13,14,22–24]. This means that sample data is always associated with covariate features that coincide in space and time, even though samples themselves have been collected at different times [14,25,26].

**Fig 1:**
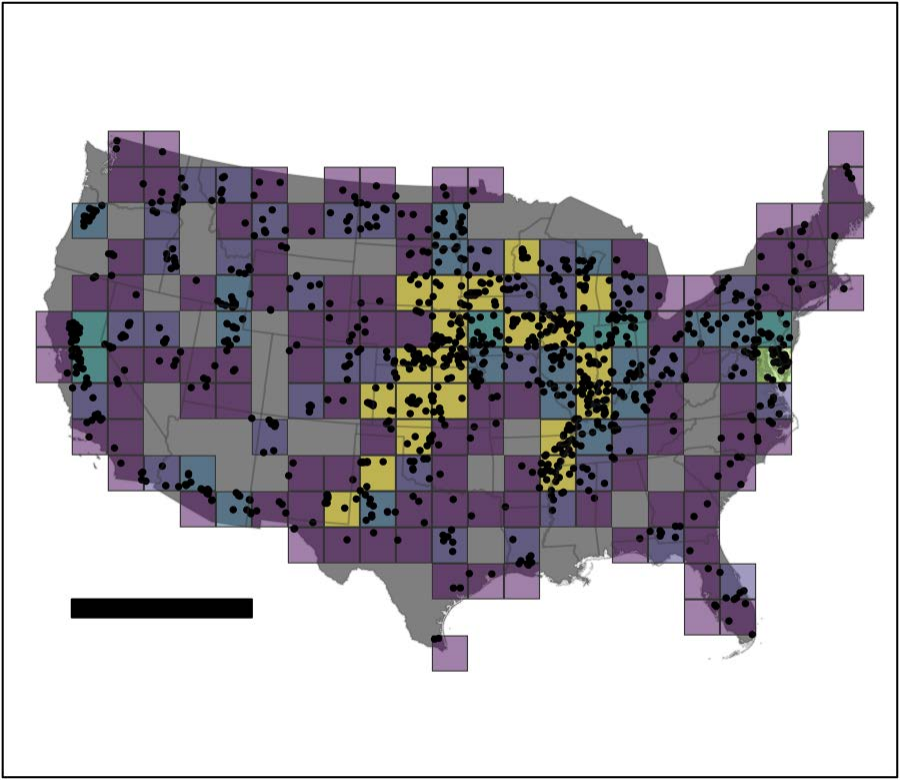
The spatial distribution of 5,230 in-situ soil samples used to develop a data-driven model of soil organic carbon as a percentage by mass in agricultural soil. Colors are proportional to the density of points within each grid cell. Scale bar is 1,000 km. State boundaries are reprinted from the Global Administrative Areas Database (GADM) under a CC BY license with permission from GADM, original copyright 2025.

Our approach uses 5,230 in-situ soil samples collected within 410 agricultural fields (Fig 1; Table 1). Most of these samples (4,260) were collected within actively cultivated, conventional row-crop agriculture in the states of Arkansas, Colorado, Illinois, Iowa, Kansas, Minnesota, Nebraska, New Mexico, Oklahoma, South Dakota, Texas, and Wisconsin during the years 2020 and 2021. The remaining 970 samples were collected in 2010 and 2011 under the USDA Rapid Carbon Assessment (RaCA) program [3; S1 Table]. At the time of collection, samples represented corn (2,249), soybeans (1,342), sorghum (400), and alfalfa (253), with the remaining 1,239 samples among less common conditions with < 100 samples each (e.g., grassland and pasture, cotton, spring wheat, and rice; Table 2). Crop types were identified using the United States Department of Agriculture (USDA) Cropland Data Layer (CDL) associated with the calendar year of sample collection. Measurements in 2020 and 2021 were acquired at randomly selected locations in fields that could be accessed before the growing season or after harvest, subject to the constraint that no sample location was within 5 m of a field boundary. The data set consists of 4,168 measurements in the 0–5 cm depth range, and 1,062 measurements of 0–30 cm. All samples were shipped to an analytical laboratory where SOC was determined as a percentage by mass using the method of dry combustion [27]. Most samples represent Mollisol soils (3,478), followed by Alfisols (913), Entisols (318), Aridosols (227) and Inceptisols (123). The remaining samples are among soil orders with < 100 samples each (Table 2). Soil orders were identified using the USDA National Resource Conservation Service (NRCS) Soil Survey Geographic Database (SSURGO).

**Table 1:** The number of in-situ soil samples in 8 cropland classes in CONUS. Crop classification is from the USDA CDL during the calendar year of sample collection.

	Alfalfa	Corn	Cotton	Grass/Pasture	Sorghum	Soybeans	Winter Wheat	Other	Total
Alabama	0	0	1	0	0	1	0	1	**3**
Arizona	4	2	11	0	0	0	0	9	**26**
Arkansas	0	131	26	1	0	37	0	35	**230**
California	4	1	2	2	0	0	2	49	**60**
Colorado	64	28	0	2	5	0	10	51	**160**
Connecticut	0	0	0	0	0	0	0	1	**1**
Delaware	0	4	0	0	0	5	0	1	**10**
Florida	0	0	0	3	0	0	0	10	**13**
Georgia	0	2	5	0	0	0	0	1	**8**
Idaho	6	4	0	6	0	0	4	10	**30**
Illinois	0	138	0	3	0	106	0	5	**252**
Indiana	0	20	0	2	0	14	1	3	**40**
Iowa	0	108	0	2	0	214	0	1	**325**
Kansas	0	224	0	3	319	145	172	6	**869**
Kentucky	0	10	0	11	0	7	0	4	**32**
Louisiana	0	0	0	1	0	2	0	5	**8**
Maine	0	0	0	0	0	0	0	5	**5**
Maryland	0	5	0	2	0	3	0	8	**18**
Massachusetts	0	0	0	0	0	0	0	1	**1**
Michigan	1	12	0	1	0	4	2	6	**26**
Minnesota	3	221	0	1	0	253	0	7	**485**
Mississippi	0	0	3	1	0	11	0	2	**17**
Missouri	0	10	2	2	0	11	0	2	**27**
Montana	4	0	0	4	0	0	1	19	**28**
Nebraska	0	811	0	7	0	371	3	4	**1196**
Nevada	11	0	0	5	0	0	0	9	**25**
New Hampshire	0	1	0	0	0	0	0	0	**1**
New Jersey	0	2	0	0	0	1	0	2	**5**
New Mexico	51	30	2	3	0	0	2	90	**178**
New York	1	5	0	0	0	0	1	7	**14**
North Carolina	0	1	1	2	0	3	0	2	**9**
North Dakota	1	3	0	7	0	7	0	7	**25**
Ohio	0	6	0	2	0	7	0	1	**16**
Oklahoma	1	102	0	0	7	2	30	26	**168**
Oregon	6	0	0	2	0	0	1	13	**22**
Pennsylvania	3	9	0	0	0	4	0	11	**27**
Rhode Island	0	0	0	0	0	0	0	0	**0**
South Carolina	0	1	1	0	0	0	0	3	**5**
South Dakota	1	133	0	3	0	7	1	8	**153**
Tennessee	0	3	3	1	0	4	0	2	**13**
Texas	1	35	23	4	69	0	42	11	**185**
Utah	14	3	0	0	0	0	0	4	**21**
Vermont	1	3	0	0	0	0	0	2	**6**
Virginia	0	4	4	1	0	2	0	1	**12**
Washington	0	0	0	2	0	0	3	8	**13**
West Virginia	0	2	0	1	0	1	0	0	**4**
Wisconsin	73	174	0	6	0	120	1	72	**446**
Wyoming	3	1	0	3	0	0	0	5	**12**
									
**Total**	**253**	**2,249**	**84**	**96**	**400**	**1,342**	**276**	**530**	**5,230**

**Table 2:** The number of in-situ soil samples in 8 cropland classes and 10 soil orders in CONUS. Crop classification is from the USDA CDL during the calendar year of sample collection. Soil orders are from the USDA-NCSS soil survey data (SSURGO).

	Alfisols	Andisols	Aridisols	Entisols	Histosols	Inceptisols	Mollisols	Spodosols	Ultisols	Vertisols	Total
Alfalfa	75	0	68	77	0	4	25	3	1	0	**253**
Corn	453	0	34	79	1	30	1,628	2	21	1	**2,249**
Cotton	23	0	5	11	0	7	17	0	12	9	**84**
Grass/Pasture	29	0	7	9	0	7	35	2	5	2	**96**
Sorghum	10	0	0	4	0	0	386	0	0	0	**400**
Soybeans	175	0	0	27	4	28	1,072	0	14	22	**1,342**
Winter Wheat	21	0	3	14	0	7	230	1	0	0	**276**
Other	127	1	110	97	2	40	85	8	23	37	**530**
											
**Total**	**913**	**1**	**227**	**318**	**7**	**123**	**3,478**	**16**	**76**	**71**	**5,230**

### 2.2. Developing the covariate data set

Predictors include features related to climate, topography, and biophysically meaningful variables derived from optical remote sensing that are correlated with soil properties and formation [12,14,28]. Here we identified 90 features using data products related to climate and weather, soil properties and topography, land use, vegetation, management, and other characteristics (S2 Table).

Previous work demonstrates that combinations of long-term climate variables, topography and optical remote sensing are predictive of organic carbon content in soils [12,14,28]. Because our analysis addresses agricultural land in particular and was designed to evaluate whether SOC content in agricultural soils can be predicted using DSM methods, we focused on the selection of covariate features known to be associated with SOC content through biological, geological, or farm-practice relationships.

Following McBratney et al. [12], we describe covariate data using the concept of resolution. Resolution is equivalent to pixel size, where native resolution refers to the source data product before reprojection or sampling, and fundamental resolution is the scale at which the feature varies in the real world. For example, the mean annual temperature from WorldClim may have a fundamental resolution of tens of kilometers, even though it is gridded at 30 arcseconds (about 1 km). Although we resampled all covariate features to 10 m resolution using a cubic spline or nearest-neighbor procedure, resampling does not change fundamental resolution. Differences in fundamental resolution among covariate features are characteristics of the environment that are consistent with the state-factor framework extended by McBratney [12] in the context of DSM. Other data-driven approaches to carbon quantification also use features at varying fundamental and native resolution [e.g., 13,24]. Hierarchical variation in fundamental resolution among features is a well-defined characteristic of data-driven analysis of environmental properties [e.g., 29,30].

We extracted values from covariate data sets at the location of each sample point. The covariate data set includes upper and lower points of soil sample measurement as quantitative predictors [22]. This allows the algorithm to use measurements collected at different depths in the soil profile to produce standardized output over the 0–30 cm depth range. For the features described below, we assign all features to SOC measurements acquired in 2020 and 2021. Data collected under the USDA RaCA program in 2010 and 2011 precede the availability of Sentinel-1 synthetic aperture radar (SAR), Sentinel-2 bottom of atmosphere surface reflectance, and Soil Moisture Active Passive (SMAP) data products. For samples acquired prior to 2020, these covariates are treated as missing values.

Missing values occur when no observation is possible or due to spatial or temporal gaps in the data record. This can occur when cloud cover results in data loss [e.g., 31]. For example, consider the following simplified feature set *x* = [1, 3, NA, 5]. This feature set represents four covariate features that could be associated with a single response. The first, second and fourth features have numerical values, but the third feature is missing, indicated by NA. One solution to missingness is to eliminate records that contain at least one missing feature value, called complete-case analysis. This is not ideal, because many records contain at least one missing value, resulting in large data losses. An alternative approach to dealing with missing data imputes missing observations using a predictive model and then analyzes the combined set of complete cases and imputed values using the same methods that are applied in a complete-case analysis [32]. Here we use a third approach developed in the context of gradient-boosted regression trees [33]. The approach identifies the default direction of the split in the decision tree for cases where the required feature is missing using non-missing records for that feature, and then selects the default direction [33].

#### 2.2.1. Long-term physical-climate proxies.

Physical-climate proxies include the WorldClim bioclimatic variables [34], which contain summaries of mean annual temperature and precipitation, temperature and precipitation seasonality and extremes at 30 arcsecond resolution. These variables represent conditions in the 1970–2000 time interval and therefore define typical climate characteristics of a given location, not the weather on any specific day. Exploratory data analysis indicated that some WorldClim variables introduced spatial mapping artifacts into model predictions. We therefore included three WorldClim bioclimatic variables as covariates: BIO1 (mean annual temperature), BIO16 (precipitation of the wettest quarter), and BIO17 (precipitation of the driest quarter).

#### 2.2.2. Short-term physical climate and weather proxies.

We generated summaries of gridded daily records from the National Centers for Environmental Prediction Climate Forecast System version 1 [NCEP CFS; 35,36]. The NCEP CFS data product is a 6 hourly summary of weather-related variables at 0.3-degree resolution (about 30 km). The variables are precipitation, soil moisture, water runoff, minimum temperature, maximum temperature, mean temperature, sensible heat net flux, downward shortwave radiation net flux, potential evapotranspiration, and transpiration. We aggregated the 6-hour data product within days using the arithmetic mean for soil moisture, mean temperature, potential evapotranspiration, transpiration, minimum temperature, maximum temperature, sensible heat net flux and downward shortwave radiation net flux, and the sum for precipitation and water runoff. Six-month and three-year summaries were generated for each variable using the arithmetic mean for the corresponding time interval prior to the target date. The target date is the date of collection of an in-situ soil sample or the date for which a prediction is desired. The NCEP data record is not available prior to April 2011. We treat all NCEP summaries as missing observations for records associated with sample dates that precede the NCEP data record (604 or 605 records, depending on the NCEP variable).

Although the NCEP summaries provide gridded temperature data at 0.3-degree resolution, variation at finer resolution influences biogeochemical processes and SOC. We therefore supplemented NCEP summaries with the 1 km MODIS land surface temperature and emissivity data product [MOD11A2 V006; 37]. MOD11A2 is an 8-day composite with 1 km native resolution. For each 1 km pixel in the MOD11A2 V006 data set, we computed the arithmetic mean in the six-month interval prior to the target date.

#### 2.2.3. Topographic and edaphic variables.

We derived surface elevation from the United States Geological Survey (USGS) 3D Elevation Program (3DEP) 10 m digital elevation model. We derived six-month and three-year soil-moisture summaries from the SMAP L3 daily 9 km soil moisture data product [38]. Summaries were the arithmetic mean within six-month and three-year windows prior to the target date. The covariate data set contained estimates of soil properties from the SoilGrids version 2.0 global 250 m data product [13]. We used gridded clay, sand and silt content as covariates.

#### 2.2.4. Synthetic aperture radar.

We used Sentinel-1 measurements derived from Level-1 Ground Range Detected (GRD) data products [39]. Sentinel-1 is a C-band radar that acquires images in all weather conditions at a variety of spatial resolutions. Here we used images acquired with dual polarizations (VH and VV) in the Interferometric Wideswath (IW) mode from ascending orbits. For each 20 m pixel in the Sentinel-1A data set, we computed the median within a six-month window prior to the target date.

#### 2.2.5. Sentinel-2 remote sensing.

We used remote sensing summaries from Sentinel-2 bottom of atmosphere surface reflectance data products [40]. For a given location there is a set of candidate Sentinel-2 observations within a time interval that could be used for training and prediction. We refer to the problem of selecting and summarizing this set of observations as image reduction, and we refer to the derived summaries as features. Engineering an image reducer to generate features requires selecting three criteria: the time interval over which the reducer will be applied, filtering that determines whether a candidate observation is included in the summary and choosing the reducing function. For example, Hansen et al. [41] applied cloud shadow and cloud cover filtering criteria to time series Landsat observations over the 2000–2012 time interval to generate a global map of forest cover change. Dvorakova et al. [42] filtered time series Sentinel-2 surface reflectance using the normalized burn ratio (NBR2) and normalized difference vegetation index (NDVI) to generate a bare soil composite. For data-driven prediction, where a model from training data is applied in the real world, the reducer must produce summaries that are spatially and temporally consistent with minimal spatial mapping artifacts. Spatial mapping artifacts can occur when there are spatially structured errors in covariate features that propagate through model predictions.

We used individual bands and derived indices from Sentinel-2 level 2 surface reflectance data products to derive features from optical remote sensing using two image reducers. These two image reducers allow the ATLAS-SOC algorithm to use covariates that represent different intervals of time. The first of these, which we call the simple reducer, computes the arithmetic median over all candidate observations for every individual location within the six-month window prior to the target date. The simple reducer thus considers the relationship between image features and SOC immediately prior to the SOC measurement. For example, if an in-situ soil sample was collected on April 9, 2021, the simple reducer would use October 9, 2020 – April 9, 2021. If a prediction is desired using a calendar date of October 16, 2021, the simple reducer would use April 16, 2021 – October 16, 2021. The second version, which we call the time-series summary reducer, computes the arithmetic median within sequential, three-month time windows over the previous 24 months. The time-series summary reducer generates statistical summaries of the land surface that are related to agricultural practices over the previous 24 months, including planting and harvest time, cover cropping, and tillage status.

We selected indices that track green and senescent vegetation cover, tillage status, and the presence of water on the land surface. Below we define each index. Band definitions for Sentinel-2 are blue (B2; 460–530 nm), green (B3; 540–0.580 nm), red (B4; 650–680 nm), near-infrared (B8; 780–890 nm), SWIR1 (B11; 1,570–1,660 nm), and SWIR2 (B12; 2,110–2,290 nm).

NDVI is correlated with cover of green vegetation and therefore distinguishes vegetated from bare-soil conditions [43]. NDVI is computed using the simple reducer and time-series summary reducer according to,


$$NDVI=\frac{NIR-red}{NIR+red}$$ (1)

The soil adjusted vegetation index (SAVI) is a modification of NDVI that corrects the index in the presence of minimal vegetation cover [44] SAVI is positively associated with vegetation cover. We computed SAVI using the simple reducer according to,


$$SAVI=1.5\times \frac{NIR-red}{NIR+red+0.5}$$ (2)

Marsett et al. [45] developed the soil adjusted total vegetation index (SATVI). The index is positively associated with green and dry vegetation. SATVI was calculated using the simple reducer according to,


$$SATVI=1.5\times \frac{SWIR1-red}{SWIR1+red+0.5}-\frac{SWIR2}{2}$$ (3)

We used the BSI to identify pixels with minimal vegetation cover and exposed soil. BSI has been used to develop bare-soil composites of agricultural land using blue, red, and shortwave-infrared reflectance [42,46]. BSI is negatively associated with vegetation cover. We computed BSI using the simple reducer and time-series summary reducer according to,


$$BSI=\frac{\left(red+SWIR1)-(NIR+blue\right)}{\left(red+SWIR1)+(NIR+blue\right)}$$ (4)

Dvorakova et al. [42] used the normalized burn ratio (NBR2) index to generate bare soil composites for prediction of organic carbon content using Sentinel-2. Larger values of NBR2 are typically associated with moist soils or crop residues [42]. The NBR2 index was calculated using the simple reducer according to,


$$NBR2=\frac{NIR-SWIR2}{NIR+SWIR2}$$ (5)

The normalized difference tillage index (NDTI) takes advantage of changes in shortwave infrared reflectance in water absorption regions to detect surface roughness associated with tillage status [47]. Larger values of the index are more likely to be associated with conventional tillage status. We computed NDTI using the simple reducer according to,


$$NDTI=\frac{SWIR1-SWIR2}{SWIR1+SWIR2}$$ (6)

The brightness index (BI) is associated with total brightness. Under bare soil conditions, brighter soils have less organic matter content than darker soils do [47]. The BI was calculated using the simple reducer,


$$BI=\sqrt{\frac{{red}^{2}+{green}^{2}}{2}}$$ (7)

The land surface water index (LSWI) is positively correlated with the total content of liquid water in vegetation and soil [48]. It was calculated using the simple reducer according to,


$$LSWI=\frac{NIR-SWIR1}{NIR+SWIR1}$$ (8)

We computed tasseled cap brightness, greenness and wetness using the Sentinel-2 coefficients [49]. Tasseled cap brightness was calculated using the simple reducer according to,


brightness=0.3510×blue+0.3813×green+0.3437×red+0.7196×NIR+0.2396×SWIR1+0.1949×SWIR2 (9)

Tasseled cap greenness was calculated using the simple reducer according to,


greenness=−0.3599×blue−0.3813×green−0.3437×red+0.7196×NIR+0.2396×SWIR1+0.2856×SWIR2 (10)

Tasseled cap wetness was calculated using the simple reducer according to,


wetness=0.2578×blue+0.2305×green+0.0883×red+0.1071×NIR−0.7611×SWIR1−0.5308×SWIR2 (11)

### 2.3. Machine learning algorithm

The algorithm is a weighted gradient-boosted regression tree [XGBoost; 33]. XGBoost was selected because tree-based regression consistently performs well on a wide-range of prediction tasks using tabular data, including SOC mapping [11,14,50]. Off-the-shelf implementations are efficient to train, able to handle missing values, and produce interpretable feature-importance metrics. The XGBoost algorithm works by assembling an ensemble of weak learners corrected iteratively to reduce bias and variance. Every weak learner is a single regression tree developed using recursive binary splitting and a user-defined objective function. Here the objective function was the mean absolute error,


$$MAE=\frac{1}{n}\sum {\mathrm{\backslash nolimits}}_{i=1}^{n}abs\left(y_{i}-{\hat{y}}_{i}\right)$$ (12)

where n is the number of training samples in the algorithm, $y_{i}$ is measured SOC as a percentage by mass for sample i, and ${\hat{y}}_{i}$ is the predicted value of SOC as a percentage by mass for sample i.

The model was fitted to all 5,230 in-situ soil samples and their associated covariates. The 4,260 data records collected in CONUS row-crop agriculture were weighted equally with a value of 1. For the remaining 970 measurements collected under the USDA RaCA program, we explored weights of 0–1 in increments of 0.05 and examined cross-validated model performance under each weighting scenario. The purpose of down weighting RaCA samples was to allow previously collected data to contribute broad spatial coverage while maintaining the importance of recently collected data in row-crop agriculture and during the period of Sentinel-2. We evaluated the ability of the model to predict SOC associated with individual in-situ soil samples and aggregated field means. Because the model was trained using physical samples, the physical-sample performance is simply the geographically-dependent cross-validated regression described below. To evaluate the performance of the model at the field level, we computed the mean SOC content using in-situ soil samples in fields that contained ≥ 5 samples (n = 165 fields and 3,285 in-situ soil samples) and the associated mean of predicted values at the same sample locations in each field.

#### 2.3.1. Cross-validation.

When models are trained using a nonrandom sample of the prediction domain, cross-validation should use geographically dependent partitions [16,51–54]. The idea is to understand how the algorithm will perform when transferred to a geographic subset of the prediction domain that is different from training data. We used individual units of land management (agricultural fields) as cross-validation folds. Most samples in our data collected under the USDA RaCA program are spatially isolated (mean nearest-neighbor distance = 30.6 km, range = 0.3–198.1 km). In contrast, new measurements collected in 2020 and 2021 were acquired on individual fields with multiple in-situ soil samples (mean = 10.4 samples per field, range = 3–133).

We evaluated model performance using the intercept and slope of the cross-validated linear regression, the root mean squared error (RMSE), and the coefficient of determination (*R*^2^). The RMSE was computed using,


$$RMSE=\sqrt{\frac{1}{n}\sum {\mathrm{\backslash nolimits}}_{i=1}^{n}{\left(y_{i}-{\hat{y}}_{i}\right)}^{2}}$$ (13)

where $y_{i}$ indicates a measurement of SOC using physical soil sampling, and ${\hat{y}}_{i}$ is the predicted value derived from machine-learning. The coefficient of determination was computed according to,


$$R^{2}=\frac{{SS}_{total}-{SS}_{residual}}{{SS}_{total}}$$ (14)

where ${SS}_{total}$ and ${SS}_{residual}$ are the total and residual sums of squares from the cross-validated regression, respectively. Because SOC as a percentage by mass is bounded at 0 and 100, we assumed a truncated Gaussian error term when computing the cross-validated regression. We quantified whether model performance varied with depth of the soil measurement using indicator-variables regression [55,56]. The regression model was,


$$y_{i}=b_{0}+b_{1}X_{1}+b_{2}X_{2}+b_{3}X_{1}X_{2}+\in_{i}$$ (15)

where $X_{1}$ is the predicted value derived from machine-learning in units of percentage-by-mass and $X_{2}$ is binary indicator variable that dictates whether $y_{i}$ was measured over the 0–5 ($X_{2}=0$) or 0–30 ($X_{2}=1$) cm depth range. The parameter $\in_{i}$ is a Gaussian error truncated over the range 0–100. When the value of $X_{2}=0$, the equation collapses to the ordinary linear regression on samples over the 0–5 cm depth range. When $X_{2}=1$, $b_{2}$ and $b_{3}$ test the null hypothesis that the cross-validated intercept and slope are equivalent between 0–5 and 0–30 cm depth ranges, respectively. The parameters $b_{2}$ and $b_{3}$ are the change in the intercept and slope for the case where $X_{2}=1$.

#### 2.3.2. Hyperparameter tuning.

XGBoost supports numerous hyperparameters that can be defined prior to fitting the model. These hyperparameters can be assigned default values or optimized in a process called hyperparameter tuning. Here we tuned five hyperparameters: the number of trees in the ensemble, the learning rate, the proportion of columns used to identify the split at each node, the proportion of samples used by each tree, and the maximum depth of each tree. We used Bayesian optimization to identify hyperparameter values [57]. We selected values that resulted in the smallest sample-level mean absolute error using the cross-validation described above.

### 2.4. Evaluation of publicly available SOC data products

Our objective is to produce a DSM algorithm that can predict SOC as a percentage by mass in North American agricultural land. However, we also need to determine whether existing data products accurately estimate field-level SOC as a percentage by mass. Although existing publicly available maps of SOC that include CONUS agricultural land were not designed for agricultural land in particular, they might be useful to VCM programs for establishing baselines in carbon removal projects, or for initialization of biogeochemical simulations of SOC fluxes. However, the accuracy of these data products in agricultural land in particular has not been assessed. These data products are generally developed using legacy soil sample data. Validating them against recent soil samples provides a useful assessment of their real-world applicability in current agricultural conditions. We compared four publicly available SOC data products to independent measurements of SOC as a percentage by mass.

#### 2.4.1. SoilGrids version 2.0.

SoilGrids version 2.0 is a global 250 m data product that contains estimates of SOC as a percentage by mass at multiple soil depths [13]. The SoilGrids 2.0 data product was developed using a Quantile Random Forest algorithm trained on about 240,000 observations worldwide and > 400 environmental covariates. Covariates included climate and temperature, geology and landcover, vegetation indices and raw measurements from the Landsat and MODIS sensors. The SoilGrids 2.0 data set contains predictions of SOC and other properties within six depth intervals. We used predictions from 0–5, 5–15 and 15–30 cm beneath the soil surface to derive an estimate of SOC within the top 30 cm of soil. We did this by computing a weighted average, where the weight applied to each depth interval was proportional to the range of that interval over 0–30 cm. For example, the weights applied to 0–5, 5–15 and 15–30 cm were 1/6, 1/3, and 1/2.

#### 2.4.2. Soil property maps at 100 m resolution.

Ramcharan et al. [58] generated 100 m resolution soil property and class maps using an approach similar to Poggio et al. [13]. The approach differs by integrating multiple soil data sets within a common analytical framework and by predicting SOC at specific depths beneath the soil surface, rather than over depth ranges. Soil data sets include the National Cooperative Soil Survey Characterization Database, the National Soil Information System, and measurements from the USDA RaCA program. We used linear interpolation among SOC predictions at 0 cm (surface), 5 cm, 15 cm and 30 cm to produce an SOC profile with 1 cm increments over the 0–30 cm depth range. We then computed the unweighted arithmetic average over all 30 increments to produce a single estimate for the top 30 cm of soil in every 100 m pixel.

#### 2.4.3. POLARIS.

Chaney et al. [59] remapped the Soil Survey Geographic Database (SSURGO) at 30 m resolution using machine learning and environmental covariates (POLARIS). The POLARIS data product does not contain estimates of SOC as a percentage by mass. We converted POLARIS estimates of organic matter within three depth ranges to SOC as a percentage by mass using the van Bemmelen factor of 0.58. Some recent work has challenged whether a single value can be used to convert organic matter into SOC [e.g., 60,61]. We considered values other than 0.58 to determine whether alignment between POLARIS data product and in-situ soil samples was sensitive to the conversion factor used (S1 Appendix). We used predictions from 0–5, 5–15 and 15–30 cm beneath the soil surface to derive an estimate of SOC within the top 30 cm of soil using the same weighted average procedure applied to the SoilGrids version 2.0 data product described in 2.4.1.

#### 2.4.4. Harmonized world soil database version 2.0.

The Harmonized World Soil Database version 2.0 (HWSD2) is a 2023 compilation of national soil surveys and legacy maps distributed as a global 30-arc-second raster [about 1 km resolution, 62]. Each pixel (called a soil mapping unit in HWSD2) is linked to attribute tables that report soil properties for seven depth layers. Because a given mapping unit can contain > 1 soil unit, each of which has distinct properties, we first calculated area-weighted SOC for each mapping unit. The area weights for each mapping unit were based on the SHARE percentage, which is the area of each soil mapping unit contained within each soil unit. Negative values in the SOC variable were ignored in subsequent analysis. To estimate mean SOC as a percentage by mass in the top 30 cm, we combined the 0–20 cm layer (D1) with half of the 20–40 cm layer (D2) using weights of 2/3 and 1/3, respectively.

#### 2.4.4. Extracting the field mean from publicly available SOC data products.

We calculated the field mean SOC over the 0–30 cm depth range using each publicly available SOC data product within each of 165 fields with ≥ 5 in-situ soil samples. We did this by re-projecting field polygons from the World Geodetic System 1984 coordinate reference system (EPSG:4326) to the native projection associated with each data product. For each of the four data sets, we extracted pixels that intersected the field geometry. Because some pixels were on boundary edges, we calculated the proportion, $w_{i},$ of each pixel, $x_{i}$, that was contained within field *j,* and used these proportions to compute the weighted mean ${SOC}_{j}$ as a percentage by mass according to,


$${SOC}_{j}=\frac{\sum_{i=1}^{n}w_{i}x_{i}}{\sum_{i=1}^{n}w_{i}}$$ (16)

## 3. Results

**Fig 2:**
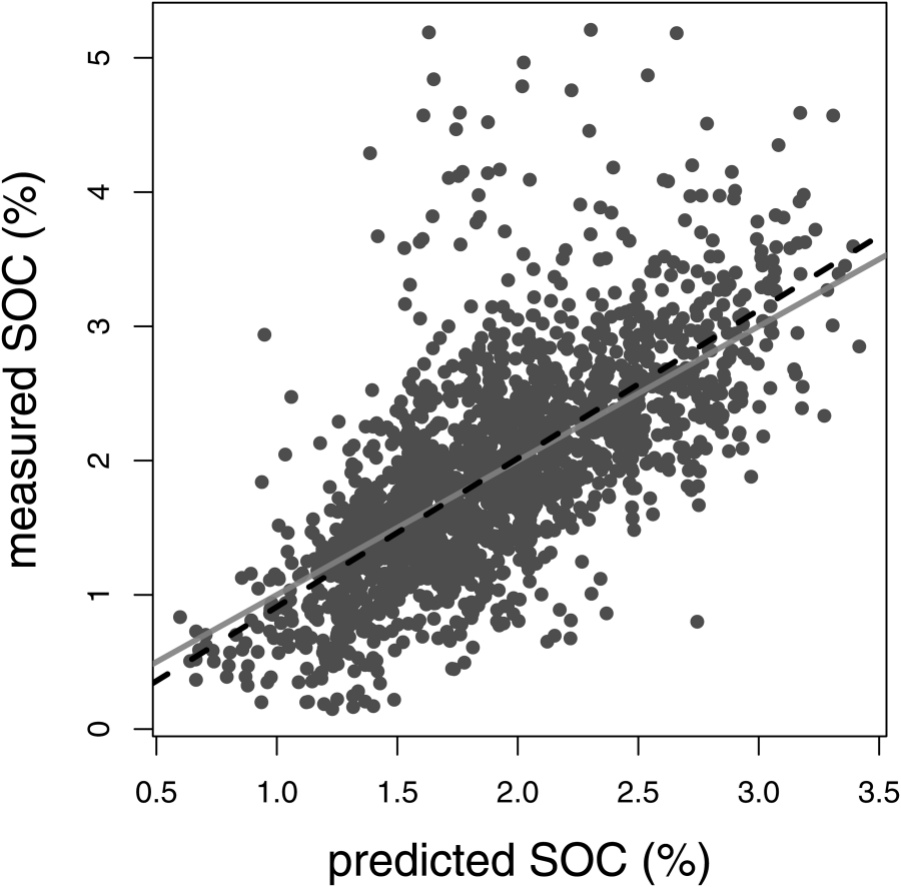
Cross-validated relationship between measured in-situ SOC as a percentage by mass and predicted values from a data-driven model in agricultural soils. Grey line is the 1:1 relationship, and the dashed line is the best-fit linear regression. This plot shows a random sample of 2,000 points for clarity, but the full data set contains 5,230 observations.

SOC can be predicted with a high degree of precision and accuracy in North American agricultural land using machine learning. Analysis of the indicator-variables regression showed that model performance was independent of the measured depth range. At the sample level, parameter estimates were $\begin{array}{c} b_{0}=-\text{0}.\text{199}(P=\text{0}.\text{959}) \end{array}$, $\begin{array}{c} b_{1}=\text{1}.\text{109}(P=\text{0}.\text{028}) \end{array}$, $\begin{array}{c} b_{2}=-\text{0}.\text{068,}(P=\text{0}.\text{978}) \end{array}$, and $\begin{array}{c} b_{3}=\text{0}.\text{010}(P=\text{0}.\text{994}) \end{array}$. We therefore dropped the indicator variable and fit the linear relationship between measured and predicted values. The intercept and slope of the cross-validated regression without indicator variables were $\begin{array}{c} b_{0}=-\text{0}.\text{249}(P<\text{0}.\text{001}) \end{array}$$\begin{array}{c} b_{1}=\text{1}.\text{130}(P<\text{0}.\text{001}) \end{array}$, respectively. The coefficient of determination was *R*^2^  = 0.487 and RMSE was 0.316 in units of percentage by mass (Fig 2).

**Fig 3:**
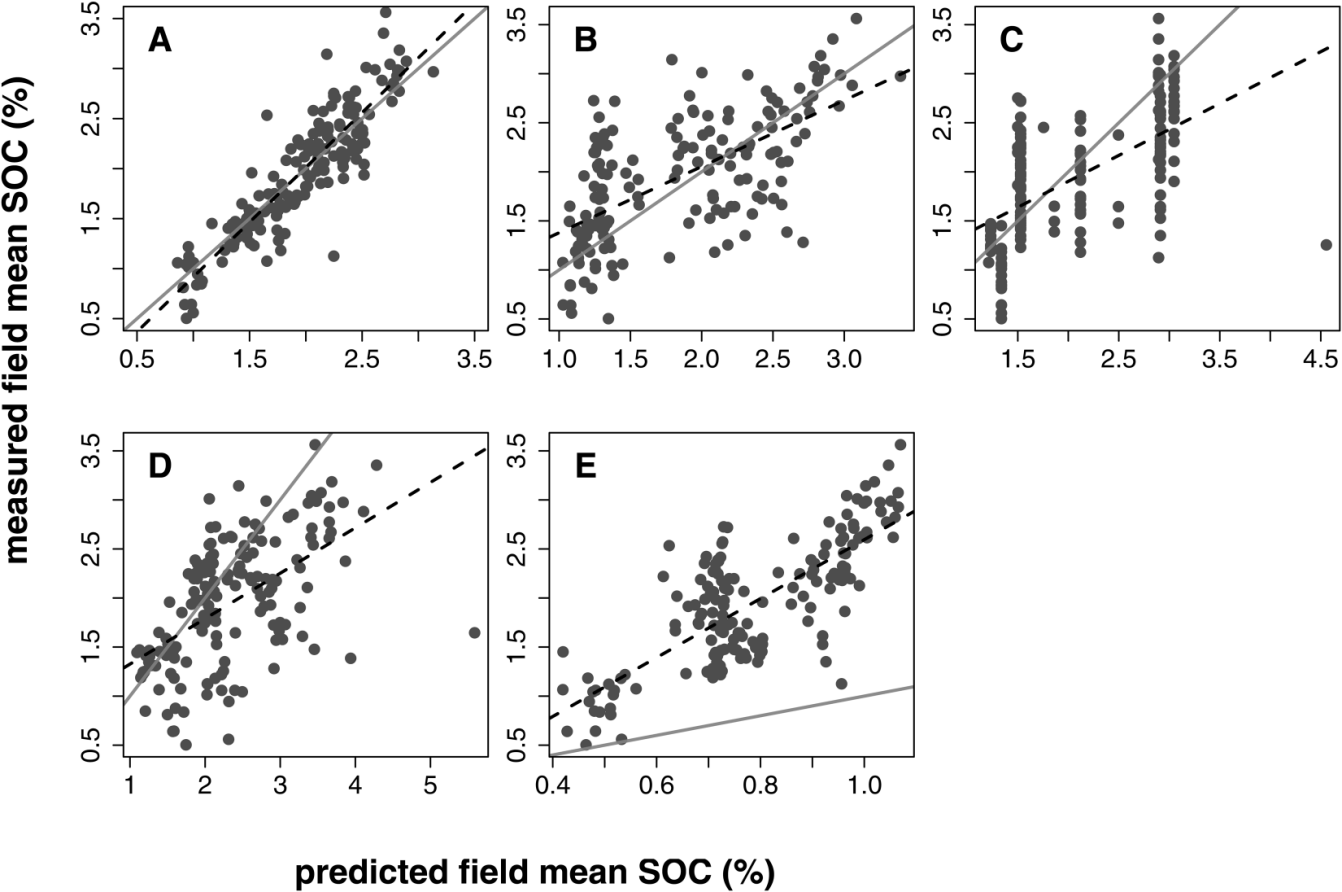
Relationships between field mean SOC and predicted values. Points are 165 fields with ≥ 5 samples (total number of samples = 3,285). (A) ATLAS-SOC (this study). (B) The 100 m soil properties and class map from Ramcharan et al. (2018). (C) the Harmonized World Soil Database version 2.0. (D) SoilGrids version 2.0. (E) POLARIS. Grey line is the one-to-one relationship, and the dashed line is the best-fit linear regression.

Aggregation to the field level demonstrated that mean predictions are accurate (Fig 3). We evaluated field-level predictions by computing the arithmetic mean in fields with ≥ 5 in-situ soil samples (n = 165 fields, 3,285 in-situ soil samples). The observed value (response variable) was the mean from sampled soil cores in the field, and the predicted value (independent variable) was the mean from pixel-level predictions at the same locations in each field. Predicted field means were always from an instance of the geographically dependent cross validation that did not contain any in-situ soil samples from the observed field. The intercept and slope of the cross-validated relationship were $b_{0}=-\text{0}.\text{179}(P=\text{0}.\text{033})$$b_{1}=\text{1}.\text{095}(P<\text{0}.\text{001})$. The coefficient of determination was *R*^2^ = 0.811, and the RMSE was 0.041 in units of percentage by mass.

Comparison of field measurements of SOC to four publicly available SOC data products showed poor ability of publicly available data products to reproduce measured values (Table 3). Ranked by RMSE, the best-performing publicly available data product was the SoilGrids version 2.0 dataset, followed by HWSD2, the 100 m resolution soil property and class map developed by Ramcharan et al. [58] and POLARIS. All of these data products exhibited substantial bias in comparison to measured values, and none of them out-performed ATLAS-SOC (Table 3; Fig 3). The SoilGrids version 2.0 data product, HWSD2, and the 100 m soil property and class map underestimated field measurements at values < 2.17%, 1.79%, and 1.61%, respectively, and overestimated field measurements at other values. The POLARIS dataset underestimated the field mean SOC as a percentage by mass in all 165 fields, although the degree of correspondence improved when a different conversion factor was used (Fig 3; S1 Appendix).

**Table 3:** Validation of mean SOC as a percentage by mass in 165 agricultural fields measured using 3,285 in-situ soil samples. $b_{0}$ and $b_{1}$ are the intercept and slope of the linear regression relating mean SOC from in-situ soil samples (response) to the predicted value from four publicly available data products and ATLAS-SOC. *R*^2^ is the coefficient of determination and RMSE is the root mean squared error.

Data product	${\mathrm{\backslash emph}b}_{0}$	${\mathrm{\backslash emph}b}_{1}$	*R* ^2^	RMSE
ATLAS-SOC (this study)	−0.179	1.095	0.811	0.041
SoilGrids version 2.0	0.705	0.675	0.412	0.148
Harmonized World Soil Database version 2	0.845	0.529	0.346	0.201
Ramcharan et al. (2018)	0.863	0.463	0.317	0.306
POLARIS	−0.409	3.004	0.585	0.831

We computed feature importance scores for every covariate in the training set. The top 20 features accounted for 68.6% of the overall importance in the data and included 7 variables from optical remote sensing, 8 weather-related proxies, 2 topographic and edaphic variables, and 2 climate variables. The most important feature type was Sentinel-2 optical remote sensing time series summaries, which collectively accounted for 28.2% of the importance in the data, followed by weather-related temperature (26.0%) and surface hydrology (11.6%; Table 4). Among feature classes, weather-related variables contributed 39.3% of the importance in the data, most of which was due to temperature (26.0%), followed by meteorology (9.5%) and precipitation (3.8%). Optical remote sensing summaries and their derivatives collectively contributed 36.3% of the importance in the data. Time series optical remote sensing summaries and their derivatives (28.2%) were about 3.5 times more important than summaries generated using the simple reducer (8.1%). Topographic and edaphic features were responsible for 18.6% of overall variable importance. Features related to surface hydrology were the most important topographic and edaphic features (11.6%), followed by the depth of the soil measurement (4.2%), silt, sand, and clay content (2.5%) and surface elevation (0.3%). Long-term physical climate proxies accounted for 5.4% of the importance in the data. Two measurements from Sentinel-1 SAR were relatively unimportant, contributing 0.5% to the overall importance in the data set (Table 4).

**Table 4:** Relative importance for 90 features used to predict organic carbon content in CONUS agricultural soils. Importance is from a gradient-boosted regression fitted to 5,230 measurements of SOC linked to covariate features and summed within feature types. For example, there are 32 time-series features from Sentinel-2 optical remote sensing whose aggregate importance is 28.2%. Totals are marginal sums within feature classes.

Feature class	Feature type	N	Importance (%)
optical remote sensing	time series summary	32	28.2
	simple summary	18	8.1
**Total**			**36.3**
weather-related	meteorology	8	9.5
	precipitation	2	3.8
	temperature	8	26.0
**Total**			**39.3**
topographic and edaphic variables	surface elevation	1	0.3
	soil (clay, silt, sand)	3	2.5
	surface hydrology	12	11.6
	depth of measurement	1	4.2
**Total**			**18.6**
long-term physical climate proxies	temperature	1	2.3
	precipitation	2	3.1
**Total**			**5.4**
Sentinel-1 SAR	synthetic aperture radar (SAR)	2	0.5

## 4. Discussion

### 4.1. Soil organic carbon measurement in support of the voluntary carbon market

VCM programs in agriculture require accurate measurement techniques that can be deployed efficiently at scale. Our analysis shows that data-driven, DSM methods can accurately predict mean SOC content in CONUS agricultural land. This analysis is based on 5,230 in-situ soil samples collected in actively cultivated, conventional row-crop agriculture, natural prairies and grasslands within 47 US states. Using a gradient-boosted regression tree and geographically dependent cross-validation, we developed a model driven by combinations of long-term climate proxies, topography and optical remote sensing variables that is both precise and accurate, overcoming challenges to widespread quantification that is necessary in support of VCM programs.

Models to predict SOC are trained using measurements from individual soil cores matched to remote sensing pixels, but VCM programs require estimates of SOC within regions. These regions could represent units of individual land management (agricultural fields) or aggregations of fields that total hundreds to thousands of hectares. Methodologies for carbon-credit generation penalize uncertainty in estimates of net GHG reductions at the scale of aggregation. Demonstrating that predictions are precise and accurate at the scale of aggregation is necessary to underpin rigorous carbon offsets in agricultural soil.

Our analysis, using a sample of 165 fields with ≥ 5 samples per field (n = 3,285 samples in total), shows that estimates of the field mean are precise and accurate when validated against fields that were excluded from model calibration (Table 3). However, comparison of field measurements of SOC to four publicly available SOC data products demonstrated poor ability of other products to reproduce measured values. Validation statistics for these data products were worse than that reported by the original authors in cases where summaries were available [13,58,59]. This is because these data products were developed to perform well throughout large regions in general, not agricultural land in particular. These products were also developed using legacy soil sample data, and it is likely that agricultural and soil conditions have changed. The data archive used to train ATLAS-SOC for this analysis includes 5,230 in-situ soil samples that were collected in 47 US states and exclusively in agricultural conditions, most of which were collected recently (S1 Table). The limited ability of publicly available data products to generalize to agricultural land in our study is an example of Simpson’s paradox, a problem in statistics where a relationship changes, or even reverses, in the presence of new variables [63]. In the context of the VCM, Simpson’s paradox underscores the importance of model validation within the domain of application. It should not be assumed, for example, that a model with generally acceptable performance in North American soils will perform well in any given subset of North America that was poorly represented by calibration data.

### 4.2. Data-driven quantification of SOC

Our analysis underscores the value of broadly distributed training data. Inclusion of geographically distributed in-situ soil samples collected under the USDA RaCA program decreased bias in the model in comparison to a version where broadly distributed data were absent (S3 Table). When the model was trained exclusively on actively cultivated, conventional row-crop agriculture in the states of Arkansas, Colorado, Illinois, Iowa, Kansas, Minnesota, Nebraska, New Mexico, Oklahoma, South Dakota, Texas, and Wisconsin, it exhibited moderate bias, as indicated by the intercept and slope of cross-validated predictions ($b_{0}=\text{0}.\text{179}$, $b_{1}=\text{0}.\text{895}$; S3 Table). But when broadly distributed in-situ soil samples were added to the training set, bias reduced (S3 Table). Geographically distributed in-situ soil samples reduced bias in the model even though they accounted for < 20% of the training set, and even though optical remote sensing and some weather variables were treated as missing values in broadly distributed data. This finding suggests that the value of geographically distributed in-situ soil samples in our analysis was primarily their contribution to resolving the relationship between SOC content, long-term physical climate proxies, and topographic and edaphic variables. This indicates that when combined with contemporary in-situ soil data, legacy soil samples can play an important role in DSM efforts.

Our findings strengthen the argument that optical remote sensing can be an important and independent source of information about SOC content in soils [10,64]. Other data-driven approaches to SOC quantification indicate that combinations of physical climate and topography are the strongest predictors of SOC content in soils of North America and China [14,65,66]. Features from optical remote sensing have been less important. The ranking of optical remote sensing in aggregate in two previous studies placed them at rank 3 or 4 out of 5 feature sets [14,66]. However, in our analysis weather-related variables and optical remote sensing summaries contributed 39.3% and 36.3% of the overall importance in the data, and 8 of the top 20 individual features were derived from optical remote sensing summaries (Table 4).

Time series summaries from Sentinel-2 were the strongest optical remote sensing predictors of SOC content (Table 4). In particular, median NDVI within three-month windows during the previous two years represented 5 of the top 20 most important features. Overall, time series summaries represented 28.2% of the importance in the data, a number 3.5 times larger than static summaries that do not consider changes in land cover over time. The sensitivity of remote sensing summaries from specific times of year suggests that land use and land cover changes are responsible for the strength of these summaries in comparison to other descriptors. One hypothesis for the importance of optical remote sensing variables in our analysis in comparison to previous work is that we increased the number of remote sensing features in comparison to other variables and used time-series feature engineering to generate predictors that are likely to be correlated with SOC content. Point-in-time optical remote sensing measurements may be less strongly correlated with SOC content than summaries that target specific land management actions.

The ranking of optical remote sensing variables in a DSM framework to quantify SOC content is important, because optical remote sensing variables are a source of information about land management, including cover cropping and reduced or no-tillage practices that are expected to impact SOC sequestration [67]. A data-driven framework that is sensitive to climate, weather, and topographic and edaphic conditions, but insensitive to variables related to land management will be able to recapitulate known physical gradients, but may not detect changes related to farm practice. More work is needed to determine whether the inclusion of practice data or biogeochemical variables in a covariate feature set – such as cover-cropping and tillage status or variables related to microbial activity – improves performance, whether data-driven calculations are sensitive to those variables, and whether direct inclusion of these variables is redundant with optical remote sensing summaries.

Interpretation of feature importance can be influenced by the number and type of variables within a covariate set and how the feature summary is generated. Our analysis contained 50 optical remote sensing features and 18 weather-related variables, 17 topographic and edaphic variables, 3 long-term physical climate proxies, and 2 measurements from Sentinel-1 SAR. This means that the algorithm is being presented with more opportunities to model the relationship using optical remote sensing than other variable types, and that overall variable importance within categories can be influenced by imbalance in the feature set. Imbalance in a covariate feature set can be desirable when an objective is to ensure sensitivity to particular kinds of features that are overrepresented.

### 4.3. Alternatives to data-driven quantification

The approach developed here was designed to overcome the challenges of efficient quantification and scale in the VCM within agricultural soils. It can be contrasted with two alternative methods, physical soil sampling and biogeochemical simulation. Physical soil sampling produces locally accurate measurements of SOC, but these measurements are costly to acquire and may demand unreasonably large sample sizes to generate precise area-based summaries required by the VCM [4,6].

Biogeochemical simulations stand on decades of research in biogeochemistry and soil science. They use local information about weather, climate, soil characteristics and farm management data to simulate interactions in soil using physics and chemistry. For example, the DeNitrification-DeComposition (DNDC) biogeochemical simulation uses climate, soil and cropping variables [68–70]. Some of these variables must be supplied by the grower (e.g., the fraction of crop residue left as stubble in the field after harvest) and other variables are difficult to measure at scale (e.g., the background NH_3_ concentration in the air). Data-driven methods from the field of DSM can help to reduce barriers to large-area estimation by allowing machine learning to model relationships between input variables and SOC content and acquiring input variables from publicly available sources. Advances by data-driven methods in comparison to traditional analyses based on physical knowledge have been achieved in other domains, including weather forecasting [71], skin cancer detection [72] and protein folding [73].

## 5. Conclusions

Data-driven calculation of SOC in agricultural soil is achievable at scale using DSM methods that are sensitive to land use and land cover change. The ATLAS-SOC algorithm developed here can predict SOC as a percentage by mass with errors that are small when evaluated at the physical sample and field mean levels. This algorithm was trained using a data set that explicitly represented row-crop agricultural land in CONUS. Field-level validation of four publicly available data products in CONUS demonstrated poor ability of publicly available SOC data products to reproduce independently measured values from in-situ soil samples.

By engineering biophysically meaningful time series features using optical remote sensing, we were able to increase sensitivity to optical remote sensing features in comparison to previous studies. Additional work is needed to determine whether optical remote sensing summaries or other variables are sensitive to land-management changes that drive SOC sequestration and baseline criteria that underpin rigorous carbon offsets.

## Supporting information

S1 Appendix Assessment of alternative values of the conversion factor used to express POLARIS SOM in units of SOC. (DOCX)

S1 Fig MAE between field mean SOC and converted POLARIS SOM. Purple line is the van Bemmelen factor, blue line is a value of 1 (no conversion), and yellow line is the value that minimizes the MAE (1.47).
(PDF)

S2 Fig Relationships between field mean SOC and predicted values. Points are 165 fields with ≥ 5 samples (total number of samples = 3,285). (A) ATLAS-SOC (this study). (B) The 100 m soil properties and class map from Ramcharan et al. (2018). (C) the Harmonized World Soil Database version 2.0. (D) SoilGrids version 2.0. (E) POLARIS (based on a conversion factor of 1.47). Grey line is the one-to-one relationship, and the dashed line is the best-fit linear regression.
(PDF)

S1 Table The number of physical soil samples collected in combinations of month and calendar year. 970 samples collected in 2010 and 2011 were acquired under the USDA Rapid Carbon Assessment Program in a wide range of land cover types, including row-crop agriculture, natural prairies and rangeland. Samples collected during 2020 and 2021 are exclusively within actively cultivated, conventional row-crop agriculture in the states of Arkansas, Colorado, Illinois, Iowa, Kansas, Minnesota, Nebraska, New Mexico, Oklahoma, South Dakota, Texas, and Wisconsin.
(DOCX)

S2 Table 90 covariate features used to predict SOC as a percentage by mass using a gradient-boosted regression tree. All features were resampled from the native resolution to 10 m using a cubic spline. Some features represent band combinations from Sentinel-2A that have different native resolutions. The number reported is the coarsest resolution among all inputs to the given feature.
(DOCX)

S3 TablePerformance of 20 candidate models used to predict SOC as a percentage by mass using physical soil samples.Weight is the per-sample weight applied to 970 previously collected and broadly distributed measurements of SOC. The remaining 4,260 measurements were weighted equally with a value of 1. The parameters $b_{0}$and $b_{1}$ are the intercept and slope from a geographically dependent cross-validation. Inclusion of broadly distributed data improved model performance (cf. the intercept and slope for weight 0.00 with all other weights). The selected model with a weight of 0.10 was retrained prior to prediction.(DOCX)
