# Supplementary material for: Digital soil mapping in support of voluntary carbon market programs in agricultural land
Source: PLoS One. 2025 Sep 2;20(9):e0327895. doi: 10.1371/journal.pone.0327895 (PMC12404560; doi:10.1371/journal.pone.0327895)
Supplement: S1 Appendix — (DOCX) [file pone.0327895.s001.docx]

**Appendix S1**

The van Bemmelen factor can be used to convert organic matter into units of SOC according to,

$SOC = SOM\times0.58$ (S1)

where SOC and SOM are soil organic carbon and soil organic matter in units of percentage by mass, and 0.58 is the van Bemmelen factor. The value of the conversion factor has been questioned, with alternative values in the range of 0.4 – 0.71 (1,2). We used the van Bemmelen factor of 0.58 to express POLARIS estimates of SOM in units of SOC so that they could be validated against independent SOC measurements obtained using dry combustion. As documented in the main text, correspondence between this converted version of the POLARIS data product and independent measurements was poor.

Here we ask whether alternative values of the conversion factor result in better correspondence. We applied conversion factors over the interval 0.01 – 2 in increments of 0.01. For each value we computed the MAE (Equation 12 in the main text) and the linear regression relationship between measured values of SOC (response variable) and converted values from POLARIS (independent variable). We do not claim that this conversion results in a physically or biologically meaningful interpretation of the POLARIS data product, but use this analysis to understand whether any rescaling of the POLARIS data product results in a better validation against independent measurements.

The relationship between MAE and the conversion factor is shown in Figure S1. A conversion factor of 1.47 minimizes the MAE. The intercept and slope of this relationship were -0.409 and 1.185, respectively. The coefficient of determination was *R*^2^ = 0.347 and the RMSE was 0.418. Note that a conversion factor of 1 results in a smaller value for the MAE than the van Bemmelen factor of 0.58 (i.e. the correspondence between POLARIS SOM and measured SOC is stronger than the correspondence between POLARIS SOC and measured SOC). We used the value of the conversion factor that minimizes the MAE to reproduce Figure 3 from the main text (Figure S2).

Figure captions

Figure S1. MAE between field mean SOC and converted POLARIS SOM. Purple line is the van Bemmelen factor, blue line is a value of 1 (no conversion), and yellow line is the value that minimizes the MAE (1.47).

Figure S2. Relationships between field mean SOC and predicted values. Points are 165 fields with ≥ 5 samples (total number of samples = 3,285). (A) ATLAS-SOC (this study). (B) The 100 m soil properties and class map from Ramcharan et al. (2018). (C) the Harmonized World Soil Database version 2.0. (D) SoilGrids version 2.0. (E) POLARIS (based on a conversion factor of 1.47). Grey line is the one-to-one relationship, and the dashed line is the best-fit linear regression.

**References**

1. Minasny B, McBratney AB, Wadoux AMJC, Akoeb EN, Sabrina T. Precocious 19th century soil carbon science. Geoderma Regional. 2020 Sep;22:e00306.

2. Pribyl DW. A critical review of the conventional SOC to SOM conversion factor. Geoderma. 2010 May;156(3–4):75–83.
