# Supplementary figures and images for: Digital soil mapping in support of voluntary carbon market programs in agricultural land
Source: PLoS One. 2025 Sep 2;20(9):e0327895. doi: 10.1371/journal.pone.0327895 (PMC12404560; doi:10.1371/journal.pone.0327895)

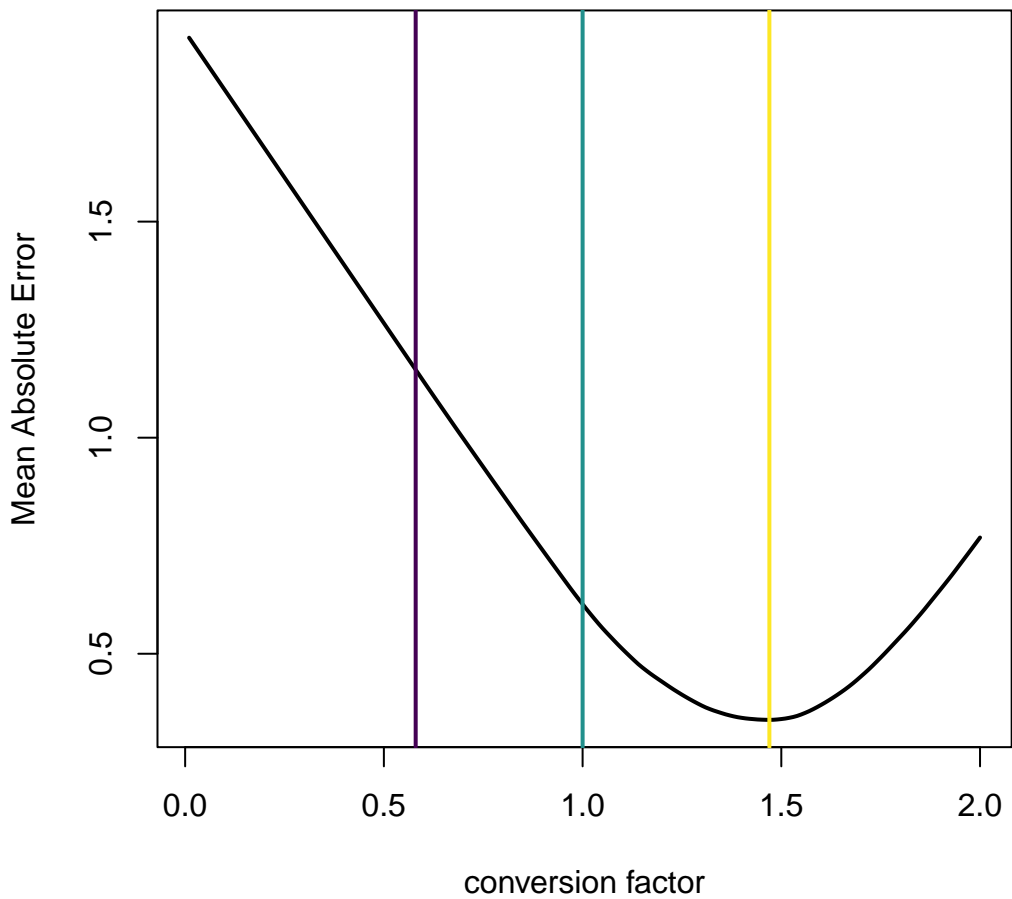

Supplement: S1 Fig — Purple line is the van Bemmelen factor, blue line is a value of 1 (no conversion), and yellow line is the value that minimizes the MAE (1.47). (PDF) [file pone.0327895.s002.pdf]

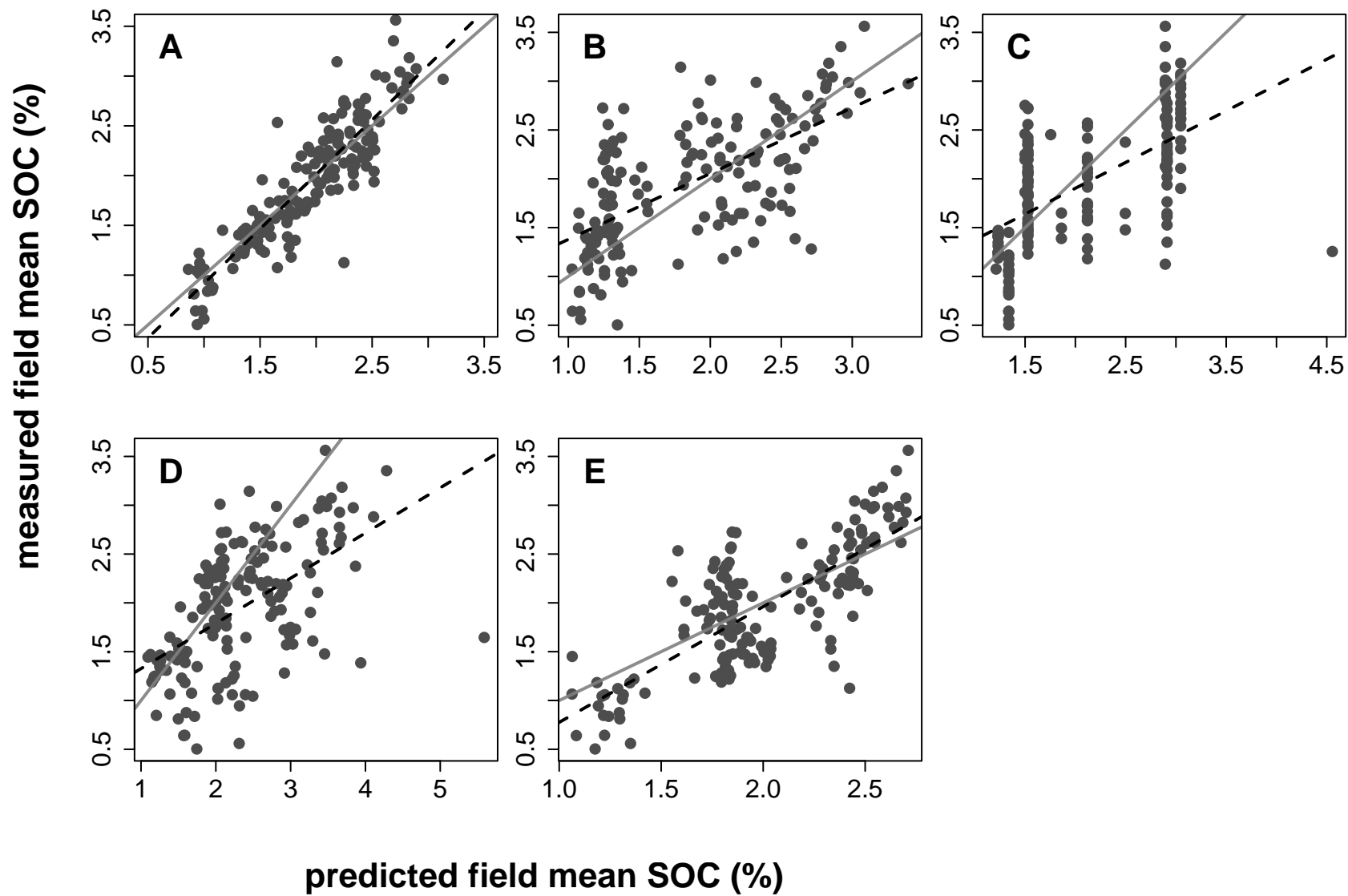

Supplement: S2 Fig — Points are 165 fields with ≥ 5 samples (total number of samples = 3,285). (A) ATLAS-SOC (this study). (B) The 100 m soil properties and class map from Ramcharan et al. (2018). (C) the Harmonized World Soil Database version 2.0. (D) SoilGrids version 2.0. (E) POLARIS (based on a conversion factor of 1.47). Grey line is the one-to-one relationship, and the dashed line is the best-fit linear regression. (PDF) [file pone.0327895.s003.pdf]
