# Supplementary material for: Digital soil mapping in support of voluntary carbon market programs in agricultural land
Source: PLoS One. 2025 Sep 2;20(9):e0327895. doi: 10.1371/journal.pone.0327895 (PMC12404560; doi:10.1371/journal.pone.0327895)
Supplement: S1 Table — 970 samples collected in 2010 and 2011 were acquired under the USDA Rapid Carbon Assessment Program in a wide range of land cover types, including row-crop agriculture, natural prairies and rangeland. Samples collected during 2020 and 2021 are exclusively within actively cultivated, conventional row-crop agriculture in the states of Arkansas, Colorado, Illinois, Iowa, Kansas, Minnesota, Nebraska, New Mexico, Oklahoma, South Dakota, Texas, and Wisconsin. (DOCX) [file pone.0327895.s004.docx]

|  |  |  |  |  |  |  |  |  |  |  |  |  |  |
| --- | --- | --- | --- | --- | --- | --- | --- | --- | --- | --- | --- | --- | --- |
| Table S1. The number of physical soil samples collected in combinations of month and calendar year. 970 samples collected in 2010 and 2011 were acquired under the USDA Rapid Carbon Assessment Program in a wide range of land cover types, including row-crop agriculture, natural prairies and rangeland. Samples collected during 2020 and 2021 are exclusively within actively cultivated, conventional row-crop agriculture in the states of Arkansas, Colorado, Illinois, Iowa, Kansas, Minnesota, Nebraska, New Mexico, Oklahoma, South Dakota, Texas, and Wisconsin. | | | | | | | | | | | | | |
|  |  |  |  |  |  |  |  |  |  |  |  |  |  |
|  | Jan | Feb | Mar | Apr | May | Jun | Jul | Aug | Sep | Oct | Nov | Dec | **Total** |
| 2010 | 0 | 0 | 0 | 0 | 0 | 0 | 1 | 2 | 32 | 122 | 182 | 52 | **391** |
| 2011 | 27 | 45 | 134 | 112 | 125 | 61 | 35 | 20 | 12 | 3 | 2 | 3 | **579** |
| 2020 | 0 | 0 | 0 | 571 | 296 | 0 | 0 | 0 | 0 | 20 | 1048 | 81 | **2016** |
| 2021 | 0 | 0 | 483 | 1218 | 12 | 0 | 0 | 0 | 0 | 371 | 160 | 0 | **2244** |
|  |  |  |  |  |  |  |  |  |  |  |  |  |  |
| **Total** | **27** | **45** | **617** | **1901** | **433** | **61** | **36** | **22** | **44** | **516** | **1392** | **136** | **5230** |
