# Supplementary material for: Digital soil mapping in support of voluntary carbon market programs in agricultural land
Source: PLoS One. 2025 Sep 2;20(9):e0327895. doi: 10.1371/journal.pone.0327895 (PMC12404560; doi:10.1371/journal.pone.0327895)
Supplement: S2 Table — All features were resampled from the native resolution to 10 m using a cubic spline. Some features represent band combinations from Sentinel-2A that have different native resolutions. The number reported is the coarsest resolution among all inputs to the given feature. (DOCX) [file pone.0327895.s005.docx]

|  |  |  |  |  |
| --- | --- | --- | --- | --- |
| Table S2. 90 covariate features used to predict SOC as a percentage by mass using a gradient-boosted regression tree. All features were resampled from the native resolution to 10 m using cubic spline. Some features represent band combinations from Sentinel-2A that have different native resolutions. The number reported is the coarsest resolution among all inputs to the given feature. | | | | |
|  |  |  |  |  |
| Feature | Data source | native resolution | Category 1 | Category 2 |
| depth of soil measurement | field measurement | - | topography and edaphic | depth |
| surface elevation | USGS 3DEP | 10 m | topography and edaphic | elevation |
| land surface temperature during day | MODIS | 1 km | weather | temperature |
| land surface temperature at night | MODIS | 1 km | weather | temperature |
| downward shortwave radiation flux (three years) | NCEP | 0.3 degrees | weather | meteorology |
| potential evaporation rate (three years) | NCEP | 0.3 degrees | weather | meteorology |
| precipitation (three years) | NCEP | 0.3 degrees | weather | precipitation |
| sensible heat next flux (three years) | NCEP | 0.3 degrees | weather | meteorology |
| volumetric soil moisture (0.0 - 0.1 m) | NCEP | 0.3 degrees | topography and edaphic | surface_hydrology |
| volumetric soil moisture (0.1 - 0.4 m) | NCEP | 0.3 degrees | topography and edaphic | surface_hydrology |
| volumetric soil moisture (0.4 - 1.0 m) | NCEP | 0.3 degrees | topography and edaphic | surface_hydrology |
| volumetric soil moisture (1.0 - 2.0 m) | NCEP | 0.3 degrees | topography and edaphic | surface_hydrology |
| mean temperature (three years) | NCEP | 0.3 degrees | weather | temperature |
| maximum temperature (three years) | NCEP | 0.3 degrees | weather | temperature |
| minimum temperature (three years) | NCEP | 0.3 degrees | weather | temperature |
| transpiration (three years) | NCEP | 0.3 degrees | weather | meteorology |
| water runoff (three years) | NCEP | 0.3 degrees | topography and edaphic | surface_hydrology |
| downward shortwave radiation flux (six months) | NCEP | 0.3 degrees | weather | meteorology |
| potential evaporation rate (six months) | NCEP | 0.3 degrees | weather | meteorology |
| precipitation (six months) | NCEP | 0.3 degrees | weather | precipitation |
| sensible heat net flux (six months) | NCEP | 0.3 degrees | weather | meteorology |
| ncep_6mo\|soilmoist1 | NCEP | 0.3 degrees | topography and edaphic | surface_hydrology |
| ncep_6mo\|soilmoist2 | NCEP | 0.3 degrees | topography and edaphic | surface_hydrology |
| ncep_6mo\|soilmoist3 | NCEP | 0.3 degrees | topography and edaphic | surface_hydrology |
| ncep_6mo\|soilmoist4 | NCEP | 0.3 degrees | topography and edaphic | surface_hydrology |
| mean temperature (six months) | NCEP | 0.3 degrees | weather | temperature |
| maximum temperature (six months) | NCEP | 0.3 degrees | weather | temperature |
| minimum temperature (six months) | NCEP | 0.3 degrees | weather | temperature |
| transpiration (six months) | NCEP | 0.3 degrees | weather | meteorology |
| water runoff (six months) | NCEP | 0.3 degrees | topography and edaphic | surface_hydrology |
| Sentinel-1A VH polarization | Sentinel-1A | 20 m | SAR | sentinel1_sar |
| Sentinel-1A VV polarization | Sentinel-1A | 20 m | SAR | sentinel1_sar |
| Quarterly binned BSI (January - March, year 0) | Sentinel-2A | 20 m | optical | optical_quarterly |
| Quarterly binned BSI (January - March, year 1) | Sentinel-2A | 20 m | optical | optical_quarterly |
| Quarterly binned BSI (April - June, year 0) | Sentinel-2A | 20 m | optical | optical_quarterly |
| Quarterly binned BSI (April - June, year 1) | Sentinel-2A | 20 m | optical | optical_quarterly |
| Quarterly binned BSI (July - September, year 0) | Sentinel-2A | 20 m | optical | optical_quarterly |
| Quarterly binned BSI (July - September, year 1) | Sentinel-2A | 20 m | optical | optical_quarterly |
| Quarterly binned BSI (October - December, year 0) | Sentinel-2A | 20 m | optical | optical_quarterly |
| Quarterly binned BSI (October - December, year 1) | Sentinel-2A | 20 m | optical | optical_quarterly |
| Quarterly binned NDVI (January - March, year 0) | Sentinel-2A | 10 m | optical | optical_quarterly |
| Quarterly binned NDVI (January - March, year 1) | Sentinel-2A | 10 m | optical | optical_quarterly |
| Quarterly binned NDVI (April - June, year 0) | Sentinel-2A | 10 m | optical | optical_quarterly |
| Quarterly binned NDVI (April - June, year 1) | Sentinel-2A | 10 m | optical | optical_quarterly |
| Quarterly binned NDVI (July - September, year 0) | Sentinel-2A | 10 m | optical | optical_quarterly |
| Quarterly binned NDVI (July - September, year 1) | Sentinel-2A | 10 m | optical | optical_quarterly |
| Quarterly binned NDVI (October - December, year 0) | Sentinel-2A | 10 m | optical | optical_quarterly |
| Quarterly binned NDVI (October - December, year 1) | Sentinel-2A | 10 m | optical | optical_quarterly |
| Lagged BSI (0 - 3 months) | Sentinel-2A | 20 m | optical | optical_lagged |
| Lagged BSI (3 - 6 months) | Sentinel-2A | 20 m | optical | optical_lagged |
| Lagged BSI (6 - 9 months) | Sentinel-2A | 20 m | optical | optical_lagged |
| Lagged BSI (9 - 12 months) | Sentinel-2A | 20 m | optical | optical_lagged |
| Lagged BSI (12 - 15 months) | Sentinel-2A | 20 m | optical | optical_lagged |
| Lagged BSI (15 - 18 months) | Sentinel-2A | 20 m | optical | optical_lagged |
| Lagged BSI (18 - 21 months) | Sentinel-2A | 20 m | optical | optical_lagged |
| Lagged BSI (21 - 24 months) | Sentinel-2A | 20 m | optical | optical_lagged |
| Lagged NDVI (0 - 3 months) | Sentinel-2A | 10 m | optical | optical_lagged |
| Lagged NDVI (3 - 6 months) | Sentinel-2A | 10 m | optical | optical_lagged |
| Lagged NDVI (6 - 9 months) | Sentinel-2A | 10 m | optical | optical_lagged |
| Lagged NDVI (9 - 12 months) | Sentinel-2A | 10 m | optical | optical_lagged |
| Lagged NDVI (12 - 15 months) | Sentinel-2A | 10 m | optical | optical_lagged |
| Lagged NDVI (15 - 18 months) | Sentinel-2A | 10 m | optical | optical_lagged |
| Lagged NDVI (18 - 21 months) | Sentinel-2A | 10 m | optical | optical_lagged |
| Lagged NDVI (21 - 24 months) | Sentinel-2A | 10 m | optical | optical_lagged |
| simple summary blue | Sentinel-2A | 10 m | optical | optical_simple |
| simple summary BI | Sentinel-2A | 10 m | optical | optical_simple |
| simple summary BSI | Sentinel-2A | 20 m | optical | optical_simple |
| simple summary LSWI | Sentinel-2A | 20 m | optical | optical_simple |
| simple summary NBR2 | Sentinel-2A | 20 m | optical | optical_simple |
| simple summary NDTI | Sentinel-2A | 20 m | optical | optical_simple |
| simple summary SATVI | Sentinel-2A | 20 m | optical | optical_simple |
| simple summary SAVI | Sentinel-2A | 10 m | optical | optical_simple |
| simple summary brightness | Sentinel-2A | 20 m | optical | optical_simple |
| simple summarygreenness | Sentinel-2A | 20 m | optical | optical_simple |
| simple summary wetness | Sentinel-2A | 20 m | optical | optical_simple |
| simple summary NDVI | Sentinel-2A | 10 m | optical | optical_simple |
| simple summary NDWI | Sentinel-2A | 10 m | optical | optical_simple |
| simple summary green | Sentinel-2A | 10 m | optical | optical_simple |
| simple summary NIR | Sentinel-2A | 10 m | optical | optical_simple |
| simple summary red | Sentinel-2A | 10 m | optical | optical_simple |
| simple summary SWIR1 | Sentinel-2A | 20 m | optical | optical_simple |
| simple summary SWIR2 | Sentinel-2A | 20 m | optical | optical_simple |
| soil moisture (three years) | SMAP | 9 km | topography and edaphic | surface_hydrology |
| soil moisture (six months) | SMAP | 9 km | topography and edaphic | surface_hydrology |
| clay content | SoilGrids | 250 m | topography and edaphic | soilgrids |
| sand content | SoilGrids | 250 m | topography and edaphic | soilgrids |
| silt content | SoilGrids | 250 m | topography and edaphic | soilgrids |
| annual mean temperature | WorldClim | 30 arcseconds | climate | temperature |
| precipitation of wettest quarter | WorldClim | 30 arcseconds | climate | precipitation |
| precipitation of driest quarter | WorldClim | 30 arcseconds | climate | precipitation |
