# Supplementary material for: Digital soil mapping in support of voluntary carbon market programs in agricultural land
Source: PLoS One. 2025 Sep 2;20(9):e0327895. doi: 10.1371/journal.pone.0327895 (PMC12404560; doi:10.1371/journal.pone.0327895)
Supplement: S3 Table — Weight is the per-sample weight applied to 970 previously collected and broadly distributed measurements of SOC. The remaining 4,260 measurements were weighted equally with a value of 1. The parameters b0and b1 are the intercept and slope from a geographically dependent cross-validation. Inclusion of broadly distributed data improved model performance (cf. the intercept and slope for weight 0.00 with all other weights). The selected model with a weight of 0.10 was retrained prior to prediction. (DOCX) [file pone.0327895.s006.docx]

|  |  |  |  |  |  |
| --- | --- | --- | --- | --- | --- |
| Table S3. Performance of 20 candidate models used to predict SOC as a percentage by mass using physical soil samples. Weight is the per-sample weight applied to 970 previously collected and broadly distributed measurements of SOC. Remaining 4,260 measurements were weighted equally with a value of 1. The parameters $b_{0}$and $b_{1}$ are the intercept and slope from a geographically dependent cross-validation. Inclusion of broadly distributed data improved model performance (cf. the intercept and slope for weight 0.00 with all other weights). The selected model with a weight of 0.10 was retrained prior to prediction. | | | | | |
|  |  |  |  |  |  |
| Weight | $b_{0}$ | $b_{1}$ | r^2^ | RMSE | RMSE (%) |
| 0.00 | 0.179 | 0.895 | 0.488 | 0.269 | 27.0 |
| 0.05 | -0.038 | 1.023 | 0.493 | 0.310 | 29.6 |
| 0.10 | -0.032 | 1.026 | 0.509 | 0.300 | 29.1 |
| 0.15 | -0.021 | 1.018 | 0.514 | 0.298 | 29.0 |
| 0.20 | 0.027 | 0.993 | 0.500 | 0.306 | 29.3 |
| 0.25 | 0.004 | 1.002 | 0.490 | 0.312 | 29.6 |
| 0.30 | 0.005 | 1.009 | 0.511 | 0.299 | 29.0 |
| 0.35 | 0.026 | 0.986 | 0.486 | 0.314 | 29.8 |
| 0.40 | 0.054 | 0.975 | 0.504 | 0.303 | 29.2 |
| 0.45 | 0.016 | 1.001 | 0.516 | 0.296 | 28.9 |
| 0.50 | 0.001 | 1.001 | 0.506 | 0.132 | 29.2 |
| 0.55 | 0.054 | 0.981 | 0.504 | 0.302 | 29.3 |
| 0.60 | 0.02 | 0.987 | 0.506 | 0.304 | 29.2 |
| 0.65 | 0.036 | 0.996 | 0.507 | 0.302 | 29.2 |
| 0.70 | 0.075 | 0.965 | 0.505 | 0.303 | 29.2 |
| 0.75 | 0.081 | 0.969 | 0.515 | 0.303 | 29.0 |
| 0.80 | 0.056 | 0.977 | 0.491 | 0.298 | 29.6 |
| 0.85 | 0.107 | 0.955 | 0.510 | 0.311 | 29.1 |
| 0.90 | 0.077 | 0.961 | 0.512 | 0.301 | 29.0 |
| 0.95 | 0.06 | 0.973 | 0.509 | 0.299 | 29.1 |
|  |  |  |  |  |  |
